# Chemical Characterization of Phenol-Rich Olive Leaf Extract (*Olea europaea* L. cv. Ogliarola) and Its Neuro-Protective Effects on SH-SY5Y Cells from Oxidative Stress, Lipid Peroxidation, and Glycation

**DOI:** 10.3390/foods15010043

**Published:** 2025-12-23

**Authors:** Maria Giovanna Rizzo, Benedetta Pizziconi, Kristian Riolo, Giovanna Cafeo, Alessia Giannetto, Marina Russo, Caterina Faggio, Laura Dugo

**Affiliations:** 1 Department of Chemical, Biological, Pharmaceutical and Environmental Sciences (ChiBioFarAm), University of Messina, Viale F. Stagno D’Alcontres 31, 98166 Messina, Italy; mgrizzo@unime.it (M.G.R.); kristian.riolo@unime.it (K.R.); alessia.giannetto@unime.it (A.G.); cfaggio@unime.it (C.F.); 2 Department of Science and Technology for Sustainable Development and One Health, Università Campus Bio-Medico di Roma, Via Alvaro del Portillo 21, 00128 Roma, Italy; benedetta.pizziconi@unicampus.it (B.P.); l.dugo@unicampus.it (L.D.); 3 Messina Institute of Technology c/o Department of Chemical, Biological, Pharmaceutical and Environmental Sciences, Former Veterinary School, University of Messina, Viale G. Palatucci Snc, 98168 Messina, Italy; giovanna.cafeo@unime.it; 4 Department of Eco-Sustainable Marine Biotechnology, Stazione Zoologica Anton Dohrn, 80121 Naples, Italy

**Keywords:** olive leaf extract, pruning waste, HPLC-PDA/MS, phenols, oleuropein, oxidative stress, lipid peroxidation, advanced glycation end product (AGE), SH-SY5Y cell, neuro-inflammation

## Abstract

Olive leaf phenols are recognized for their antioxidant and anti-inflammatory properties. A hydroalcoholic extract of *Olea europaea* L. cv. Ogliarola leaves was recovered with an ultrasound-assisted extraction using green solvents. Phenol content was investigated by means of liquid chromatography coupled with photodiode array and mass spectrometer detectors. Extract cytotoxicity was determined in SH-SY5Y neuroblastoma cells by the MTT assay to establish non-cytotoxic concentrations. The effects of the extract under lipopolysaccharide-induced conditions were investigated by assessing oxidative stress and lipid peroxidation through malondialdehyde quantification using the thiobarbituric acid assay. Antiglycation capacity was examined with a BSA methylglyoxal model. In parallel, quantitative real-time PCR was employed to assess the modulation of inflammation- and oxidative stress-related genes (TLR4, NF-κB, IL-6, IL-8, Nrf2, and HO-1), providing molecular insights into the extract’s bioactivity. The extract did not exert cytotoxic effects at the selected concentrations and with modulated oxidative stress, lipid peroxidation, protein glycation, and gene expression profiles associated with inflammatory and redox pathways in neuronal cells. These data demonstrated that olive leaf extract, rich in phenols, influenced multiple biochemical and molecular endpoints relevant to neuronal physiology, supporting its potential application as a nutraceutical ingredient for the modulation of oxidative and glycation-related processes.

## 1. Introduction

The focus of research in the field of food chemistry has historically been on the bioactive molecules found in native plants of the Mediterranean basin, which are of significant interest to researchers in this area due to their abundance [1,2,3]. Olive tree (*Olea europaea* L.) represents one of the most emblematic species of the Mediterranean region [4] of which leaves are the most abundant agro-industrial byproducts generated from its cultivation [5]. Olive leaves have recently attracted attention as rich sources of bioactive phenolic compounds such as oleuropein, hydroxytyrosol, verbascoside, tyrosol, and flavonoids including luteolin and apigenin derivatives, which contribute to their strong antioxidant, anti-inflammatory and antiglycation potential, making them the perfect candidates for nutraceutical formulations [6]. Their recovery aligns with circular economy strategies aimed at reducing waste and enhancing the sustainability of Mediterranean olive production systems, transforming agro-industrial byproducts in valuable sources of health-promoting bioactive matrices [7].

The chemical composition of olive leaves is strongly influenced by the cultivar, agronomic conditions, season and geographic origin [6]. From a proximate composition standpoint, olive leaves generally contain high levels of organic matter, amino acids, and carbohydrates, with mannitol and glucose representing the main soluble sugars, while crude proteins and lipids are typically present in lower amounts [8]. Although this general matrix composition is shared across *Olea europaea* cultivars, phenolic profiles show pronounced cultivar-dependent variability [9]. Oleuropein, hydroxytyrosol, verbascoside, and flavonoid glycosides such as luteolin-7-O-glucoside are common constituents across most cultivars; however, their relative abundance differs significantly among genotypes [8]. For instance, cultivars such as Coratina and Caiazzana are consistently reported to accumulate higher oleuropein levels [10]. In contrast, Arbequina generally displays lower total phenolic content when compared to Hoji-blanca, Arbosana and Koroneiki, reflecting its distinct metabolic and agronomic profile [11]. These compositional variations highlight the importance of cultivar selection when evaluating the biological properties of olive leaf extracts, as genotype-driven differences in phenolic profiles can significantly influence the antioxidant, antiglycation, and anti-inflammatory activity [8,9]. The *Olea europaea* L. cv. Ogliarola, a traditional Sicilian cultivar, is characterized by high phenolic content and potential health promoting properties, yet its biochemical profile and biological properties remain comparatively underexplored [12]. Understanding its cultivar-specific phytochemical features is essential for correlating composition with potential biological activity and for identifying extract profiles suitable for high-value nutraceutical applications.

Oxidative stress, glycation, and inflammation are interconnected processes that contribute to neuronal dysfunction and are implicated in neurodegenerative disorders [13]. Lipid peroxidation products such as malondialdehyde (MDA) are widely used as biomarkers of neuronal oxidative injury [14], while advanced glycation end-products (AGEs) promote neuronal cellular stress and inflammatory responses [15]. However, no integrated evaluation of oxidative stress, glycation, and inflammation has been reported for Ogliarola olive leaf extracts in neuronal models. The SH-SY5Y neuroblastoma line is one of the most widely used human-derived neuronal models to investigate cellular responses to oxidative stress, lipid peroxidation, and inflammatory signaling [16,17]. These cells express key receptors and transcriptional regulators involved in neuroinflammation, including TLR4, NF-κB [18,19], and the antioxidant response elements controlled by Nrf2/HO-1 [20,21], making them particularly suitable for mechanistic studies of redox–inflammatory crosstalk in neuronal physiology [22]. Addressing this knowledge gap is crucial for assessing their potential as multifunctional neuroprotective agents. The objective of this study was to investigate the biological actions of olive leaf extracts obtained from the Ogliarola cultivar. The olive leaves were first characterized in bioactive molecules using a liquid chromatographic system coupled with a photodiode array and mass spectrometer detectors. Then the research focused on the antioxidant, antiglycation, and anti-inflammatory properties of the extract in human SH-SY5Y neuroblastoma cells. The findings offer new insights into the molecular pathways responsible for these effects, indicating that *Olea europaea* L. cv. Ogliarola leaf extract may serve as a valuable natural source of phenolic compounds for use in functional foods and nutraceutical formulations.

## 2. Materials and Methods

### 2.1. Chemicals

Water, acetonitrile, methanol (UHPLC-MS grade, purity ≥ 99.9%), ethanol, methyl *tert*-butyl ether (MTBE), dichloromethane (gradient grade for HPLC, purity ≥ 99.9%), and formic acid were used for extraction and chromatographic analyses. For the quantification of phenols, the following standard compounds were used: apigenin, gallic acid, hydroxytyrosol, luteolin, oleuropein, verbascoside. All the chemicals used in this study were purchased from Merck Life Science (Merck KGaA, Darmstadt, Germany).

### 2.2. Samples and Sample Preparation

Olive leaves were collected from five trees of Ogliarolain February 2025 in Messina (Italy). The leaves were washed with distilled water and dried at room temperature for 15 days. Then, the dried leaves were ground using a laboratory mill and sieved to obtain particle sizes of less than 0.3 mm. Hermetically sealed bags were used to store the powder at −20 °C in the dark for 7 days before extraction procedure. The extraction of bioactive compounds was performed according to the procedure previously developed by Benincasa and co-workers [23], with some modifications. In detail, 500 mg of powder was weighed in a 15 mL falcon tube and 5 mL of ethanol/water (8:2, *v/v*) were added. Following sonication at 25 °C for 10 min in an ultrasonic bath Elmasonic P (Elma Schmidbauer GmbH, Singen, Germany) [24], the mixture was centrifuged for 5 min at 4500 rpm, using a Neya XS centrifuge (REMI Sales & Engineering Ltd., Mumbay, India). After centrifugation, the upper layer was collected. The extraction procedure was repeated three times with the same solvent amount. Finally, the three extract aliquots were combined and filtered through a 0.45 μm syringe filter.

### 2.3. Phenols Characterization by Means of HPLC-PDA-ESI/MS

The profiling of phenolic constituents was performed using a Shimadzu Nexera X2 HPLC system equipped with a photo-diode array detector (PDA, SPD-M30A) and an LCMS-2020 single quadrupole mass spectrometer (Shimadzu, Duisburg, Germany). Separation was achieved on an Ascentis Express C18 column (150 × 4.6 mm, 2.7 μm; Merck KGaA, Darmstadt, Germany), following the procedure validated by Dugo et al. [25].

Mobile phase A consisted of water containing 0.1% formic acid, while phase B was acetonitrile with 0.1% formic acid. The gradient elution program was set as follows: 10% B at 0 min; 35% B at 4 min; 47% B at 12 min; 60% B at 12.5 min; 75% B at 16 min; and 100% B at 21 min. The column temperature was maintained at 28 °C, with a flow rate of 1.0 mL/min, and an injection volume of 5 μL. PDA data were recorded across 200–700 nm, and chromatograms were processed at 280 nm.

Mass spectrometric detection was performed using an electrospray ionization (ESI) source in negative mode. MS spectra were collected in the *m*/*z* range 100–800 with an event time of 0.5 s. Interface, desolvation line, and heat block temperatures were set to 300, 280, and 300 °C, respectively, with nebulizing and drying gas flow rates of 1.5 and 5.0 L/min. Data acquisition and processing were conducted using LabSolution software version 5.95 (Shimadzu, Duisburg, Germany).

Phenolic compounds were identified by combining UV absorption features and MS signals, according to literature data and reference standard when available (please see Table S1). Quantification was carried out in PDA mode using external calibration curves in ethanol prepared for gallic acid, hydroxytyrosol, verbascoside, luteolin, apigenin, and oleuropein in the linear range of 0.5–100 mg L^−1^ (0.5, 1, 5, 10, 50, and 100 mg L^−1^). Hydroxytyrosol glucoside was quantified with hydroxytyrosol regression curve. Apigenin and luteolin calibration curves were employed to quantify their derivatives. On the other hand, oleuropein regression curve was used to characterize hydroxyoleuropein, oleoside, lucidumoside C and oleuropein glucoside. Method validation was carried out according to the method previously developed and validated by Dugo et al. [25]. Validation parameters were reported in Supplementary Material, Table S1.

### 2.4. Determination of Total Phenolic Content

The total phenolic content (TPC) of *Olea europaea* L. cv. Ogliarola leaf extract was measured using the Folin–Ciocalteu colorimetric method as described by Singleton et al. (1999) [26] with minor modifications. Briefly, 20 μL of extract were mixed with 1580 μL of methanol/water (50:50, *v*/*v*) and 100 L of Folin–Ciocalteu reagent (Thermo Fisher Scientific, Waltham, MA, USA). The mixture was incubated for 8 min at room temperature in the dark, followed by the addition of 300 μL sodium carbonate solution (0.2 g/mL) and further incubation for 2 h under the same light-protected conditions. Samples were centrifuged at 20,000× *g* for 5 min at room temperature, and 200 μL of the supernatant were transferred into a clear 96-well microplate. Absorbance was measured at 765 nm using an Infinite^®^ 200 PRO multimode microplate reader (Tecan, Männedorf, Switzerland). A calibration curve was prepared using gallic acid (50–1500 μg/mL), and TPC was expressed as mg gallic acid equivalents (mg GAE) per gram of dry extract.

### 2.5. Antiglycation Assay

The antiglycation activity of *Olea europaea* L. cv. Ogliarola leaf extract was evaluated using a BSA–methylglyoxal (MGO) in vitro model, following previously reported procedures with slight modifications [27]. BSA (4 mg/mL) and MGO (20 mM) were dissolved in phosphate-buffered saline (PBS, pH 7.4) containing 0.02% sodium azide to prevent microbial growth. The extract was added at final concentrations of 10, 20, 50, 100, and 200 μg/mL, and samples were incubated at 37 °C in the dark for 7 days. Negative controls consisted of BSA solutions without MGO.

AGE formation was assessed by measuring fluorescence at 365/440 nm using an Infinite^®^ 200 PRO multimode microplate reader (Tecan, Männedorf, Switzerland). Inhibition (%) was calculated using:I (%) = [1 − (Fextract/Fcontrol)] × 100

Where represents the fluorescence intensity of the sample treated with extract and refers to the fluorescence of the BSA–MGO system in the absence of extract.

### 2.6. Cell Culture

SH-SY5Y human neuroblastoma cells (ATCC^®^ CRL-2266, Manassas, VA, USA) were cultured in DMEM/F12 supplemented with 10% fetal bovine serum (FBS) (Merck Life Science, Milan, Italy), 1% penicillin–streptomycin (Merck Life Science, Milan, Italy), and 2 mM L-glutamine (Merck Life Science, Milan, Italy). Fetal bovine serum (FBS) was heat-inactivated at 56 °C for 30 min prior to use. Cells were maintained at 37 °C in 5% CO_2_ and subcultured twice weekly using 0.05% trypsin–EDTA [28].

### 2.7. Cytotoxicity Assessment by MTT Assay

SH-SY5Y cells were seeded in 96-well plates at 1 × 10^4^ cells/well and allowed to adhere overnight. Cells were then treated with olive leaf extract at 5, 10, 20, 50, 100, 200, and 500 μg/mL for 24 or 48 h at 37 °C and 5% CO_2_. Cell viability was assessed by adding MTT reagent (1 mg/mL; Sigma-Aldrich, St. Louis, MO, USA, 475989) for 2 h in the dark, followed by solubilization of formazan crystals in DMSO (Merck Life Science, Milan, Italy). Absorbance was read at 540 nm (Infinite^®^ 200 PRO, Tecan, Mannedorf, Switzerland). Viability was expressed relative to untreated controls. Each condition was tested in triplicate wells (technical replicates), and experiments were independently repeated at least three times using different cell passages (biological replicates) [29].

### 2.8. Cell Cultureand Induction of Inflammatory Response

SH-SY5Y cells were seeded in 12-well plates and allowed to reach confluence. Inflammation was induced by exposure to lipopolysaccharide (LPS, 1 μg/mL, *Escherichia coli* O26:B6, Merck Life Science) for 24 h. After this stimulation period, the culture medium was completely removed and cells were gently washed once with pre-warmed phosphate-buffered saline to eliminate residual LPS. Fresh complete medium containing olive leaf extract (OLE) at non-cytotoxic concentrations (10, 50, and 100 μg/mL) was then added, and cells were incubated for an additional 24 h. Control cells underwent the same medium replacement procedure but were not exposed to LPS or OLE. At the end of the OLE post-treatment period, cells were processed for RNA extraction and malondialdehyde (MDA) determination at the same experimental time-point [30]. This experimental design was intended to investigate gene-expression changes during a late phase of the LPS response after removal of the inflammatory stimulus, rather than acute TLR4/NF-κB activation. Accordingly, early signaling kinetics and direct co-treatment effects between LPS and the olive leaf extract were not addressed. Dose selection also considered morphology and time-dependent viability.

### 2.9. RNA Extraction, cDNA Synthesis and Quantitative Polymerase Chain Reaction (qPCR)

Total RNA was isolated from SH-SY5Y cells using TRIzol reagent (Thermo Fisher Scientific, USA). RNA purity and concentration were determined spectrophotometrically. After DNase treatment, 1 μg of RNA was reverse-transcribed using the iScript gDNA Clear cDNA Synthesis Kit (Bio-Rad, Hercules, CA, USA). qPCR was performed using GoTaq^®^ qPCR Master Mix (Promega, Madison, WI, USA) on a Rotor-Gene Q2 plex HRM system (Qiagen, Venlo, The Netherlands), with primers targeting IL-6, IL-8, NF-κB, TLR4, Nrf2, and HO-1 (Table 1). Reactions were run in duplicate with -RT and no-template controls. Melt-curve analysis confirmed single amplicons. Relative expression was calculated using the 2^−ΔΔCt^ method with GAPDH as the reference gene, consistent with its use in the same SH-SY5Y/LPS model [31].

### 2.10. Determination of Malondialdehyde (MDA) Levels

Lipid peroxidation was quantified by determining MDA levels using the thiobarbituric acid (TBA) assay [34]. Cell samples were mixed with 20% trichloroacetic acid (TCA) and 0.5% TBA and heated at 95 °C for 30 min to form the MDA–TBA adduct. Samples were cooled on ice and centrifuged at 10,000× *g* for 5 min. The supernatant absorbance was measured at 532 nm using a Shimadzu UV1800 spectrophotometer. MDA concentration was calculated from a standard curve (1–50 μM) and expressed as μM MDA.

### 2.11. Statistical Analysis

**Figure 1:**
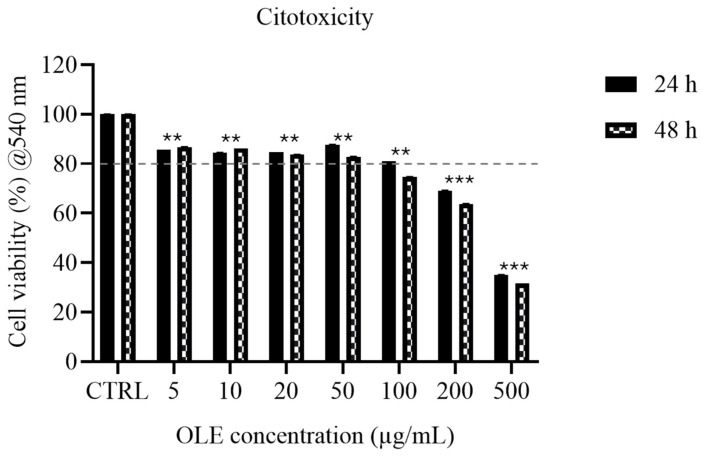
MTT assay of SH-SY5Y cells incubated to OLE (5–500 µg/mL) for 24 h (solid bars) and 48 h (hatched bars). Data are normalized to untreated control (100%). Data are presented as mean ± SD. Statistical analysis was performed using one-way ANOVA followed by Bonferroni post hoc test (** *p*-values < 0.01 *** *p*-values < 0.001). The dashed line indicates the ~80% viability value referenced in ISO 10993-5 as a screening threshold for potential overt cytotoxicity; values above this threshold do not necessarily indicate biological neutrality and were interpreted together with morphological observations.

**Figure 2:**
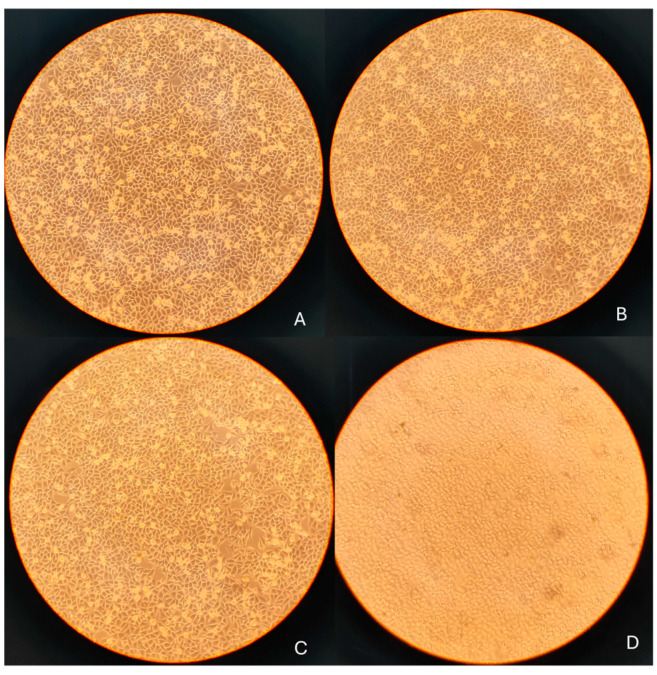
Phase-contrast images of SH-SY5Y cells following 24 h treatment with OLE: (**A**) untreated control; (**B**) 10 µg/mL; (**C**) 50 µg/mL; (**D**) 100 µg/mL. 10× objective; scale bar = 100 µm.

Statistical analyses were performed using GraphPad Prism 10.0. Data are expressed as mean ± SD of at least three independent biological experiments. For MTT viability data (Figure 1), one-way ANOVA followed by Bonferroni’s post hoc test was applied to account for multiple comparisons across extract concentrations and incubation times. The ~80% viability value was used only as a functional screening criterion to flag potential overt cytotoxicity (ISO 10993-5, as previously reported in a our published study [29]) and to guide dose selection; it was not interpreted as a threshold of biological neutrality. For gene-expression (Figures 4 and 5) and MDA data (Figure 2), one-way ANOVA followed by Tukey’s post hoc test was used. Statistical significance was set at *p* < 0.05.

## 3. Results

### 3.1. Total Phenolic Characterization and Content

As reported in Table 2, the total concentration of bioactive compounds was 106,828.3 ± 246.6 mgkg^−1^. Oleuropein is the most abundant phenolic compounds characterized by the hydroalcoholic extract of olive leaves analyzed, in accordance with previously published literature data [35]. According to Table 1, oleuropein content was about 78% of the total phenolic molecules determined by means of HPLC-PDA-ESI/MS. Other significant compounds were hydroxytyrosol and its glucoside, verbascoside, luteolin and apigenin derivatives, all contributing to the anti-inflammatory, neuroprotective, and cell-protective actions of the extract [36]. These phenolic compounds profile identify the olive leaf hydroalcoholic extract as a high-value natural product. Indeed, the presence of various phytochemicals may offer complementary or synergistic health effects that may enhance bioactivity.

The total phenolic content (TPC) of the *Olea europaea* L. cv. Ogliarola leaf extract was determined using the Folin–Ciocalteu assay. Six independent replicates of the same extract were analyzed, showing good reproducibility among measurements. The mean value was 330 mg gallic acid equivalents (GAE)/g dry weight, confirming the high phenolic content of the matrix.

Such richness in polyphenolic compounds is consistent with the phytochemical profile generally reported for olive leaf extracts and provides a biochemical rationale for their biological activity. In particular, polyphenols are well known to modulate oxidative stress and inflammatory responses, thus supporting the subsequent investigation of the antioxidant, antiglycation, and anti-inflammatory properties of the extract. It is important to note that the sum of the phenolic compounds structurally identified by HPLC-PDA-ESI/MS (≈106.8 mg/g extract) represents only a fraction of the total phenolic content (TPC = 330 mg GAE/g extract) determined by the Folin–Ciocalteu assay. This discrepancy is expected, as the Folin–Ciocalteu reagent responds not only to the quantified individual phenolics but also to a broader range of reducing species naturally present in the extract. These may include polymeric phenols, low-molecular-weight phenolic acids not detectable under our chromatographic conditions, and other non-phenolic reducing constituents. Therefore, the TPC value reflects the overall reducing capacity of the extract, while the HPLC-based analysis accounts only for compounds that could be individually separated and structurally assigned. Taken together, these data indicate that approximately one-third of the Folin-responsive material was identified at the molecular level, consistent with the known analytical behavior of complex botanical matrices.

### 3.2. Antiglycation Activity

The antiglycation activity of *Olea europaea* L. cv. Ogliarola leaf extract was assessed after 7 days of incubation of the BSA–MGO system in the presence *of* increasing concentrations of OLE (10–200 µg/mL). The AGEs formation inhibition of OLE was normalized for the positive control BSA-MGO system alone. A clear concentration-dependent trend was observed. The percentage inhibition values were 16.39%, 12.32%, 24.88%, 16.48%, and 31.5% for 10, 20, 50, 100, and 200 µg/mL OLE,, respectively. The highest inhibition was obtained at 200 µg/mL, which produced the strongest reduction in AGEs formation. At 100 µg/mL, inhibition was moderate, although slightly lower than the peak observed at 50 µg/mL. Lower concentrations (10 and 20 µg/mL) showed moderate antiglycation effects, similar to those exerted at 100 µg/mL. Fluorescence measurements confirmed the inhibitory trend. The BSA–MGO positive control showed the highest fluorescence intensity (512.5), reflecting extensive AGEs formation, whereas BSA alone, included as negative control, displayed minimal signal (43.5). OLE treatment progressively reduced fluorescence intensities across the tested concentrations. Consistently with the inhibition data, 200 µg/mL OLE produced the lowest fluorescence value among treated samples (351), indicating the most effective suppression of AGEs formation. The 50 µg/mL also markedly lowered fluorescence (385) relative to the positive control, supporting the strong antiglycation potential of the extract. 10, 20 and 100 µg/mL OLE fluorescence intensity recorded was moderate and similar across samples (428.5, 449.3 and 428 respectively). These results demonstrate that the extract exerts a moderate antiglycation effect, in line with the established capacity of plant polyphenols to counteract protein glycation. Given the central role of AGEs in diabetes-related complications and other chronic degenerative conditions, the inhibitory activity observed highlights the potential nutraceutical relevance of olive leaves polyphenols. The antiglycation activity observed for the OLE is quantitatively consistent with previously reported data on olive leaf extracts evaluated in in vitro protein glycation models. In a recent study, polyphenol-rich olive leaf extracts tested in BSA–glucose systems reduced AGEs formation by approximately 9–28 fold, depending on concentration, whereas triterpene-enriched extracts showed a more moderate inhibition of approximately 1.5–2.5 fold [37]. In the present study, OLE inhibited AGE formation by 12–32% in the BSA–MGO model at concentrations between 10 and 200 μg/mL, with the strongest effect observed at 200 μg/mL. Although differences in glycation inducers (glucose vs. methylglyoxal), incubation conditions, and extract composition prevent a direct numerical comparison, the magnitude and concentration-dependent trend of inhibition are in line with those reported for chemically characterized olive leaf extracts. These data support the biological relevance of the antiglycation activity observed for OLE.

### 3.3. Cytotoxicity Assessment (MTT Assay)

The effect of *Olea europaea* L. cv. Ogliarola leaf extract (OLE) on SH-SY5Y cell viability was assessed using the MTT assay after 24 and 48 h of exposure (Figure 1). Cell viability was evaluated as a functional screening parameter. In line with ISO 10993-5, values below ~80% may indicate overt cytotoxicity; however, this guideline threshold does not imply biological neutrality, and statistically significant reductions above 80% may still reflect sublethal or stress-related effects. Therefore, viability data were interpreted together with morphological observations. It should be noted that statistical significance versus control indicates a measurable change in metabolic activity but does not necessarily imply cytotoxicity. At 24 h, OLE concentrations ranging from 5 to 100 µg/mL-maintained cell viability above the cytotoxicity threshold. Specifically, viability values were 85.62% at 5 µg/mL, 84.47% at 10 µg/mL, 84.74% at 20 µg/mL, 87.70% at 50 µg/mL, and 80.98% at 100 µg/mL. In contrast, higher concentrations resulted in a clear dose-dependent reduction in viability, which decreased to 69.06% at 200 µg/mL and 35.09% at 500 µg/mL. After 48 h of treatment, a further reduction in viability was observed, indicating a time-dependent effect. Concentrations of 5–50 µg/mL remained non-cytotoxic, with viability values of 86.77%, 86.23%, 83.72%, and 82.89%, respectively. However, at 100 µg/mL, viability dropped below the 80% threshold to 74.59%, while 200 µg/mL and 500 µg/mL produced marked cytotoxicity, reducing viability to 63.78% and 31.54%, respectively. Overall, OLE was within the no-overt-cytotoxicity range up to 100 µg/mL at 24 h, whereas the safe concentration range decreased to ≤50 µg/mL at 48 h. These results highlight both a dose- and time-dependent cytotoxic effect, consistent with the biphasic behavior of polyphenol-rich extracts, which are generally well tolerated at low-to-moderate concentrations but may exert cytotoxicity at higher doses or after prolonged exposure. This definition of within the no-overt-cytotoxicity range ranges provides a functional criterion for selecting concentrations suitable for subsequent mechanistic studies, distinguishing biological modulation from effects potentially influenced by reduced cell viability.

### 3.4. Morphological Assessment Prior to qRT-PCR

**Figure 3:**
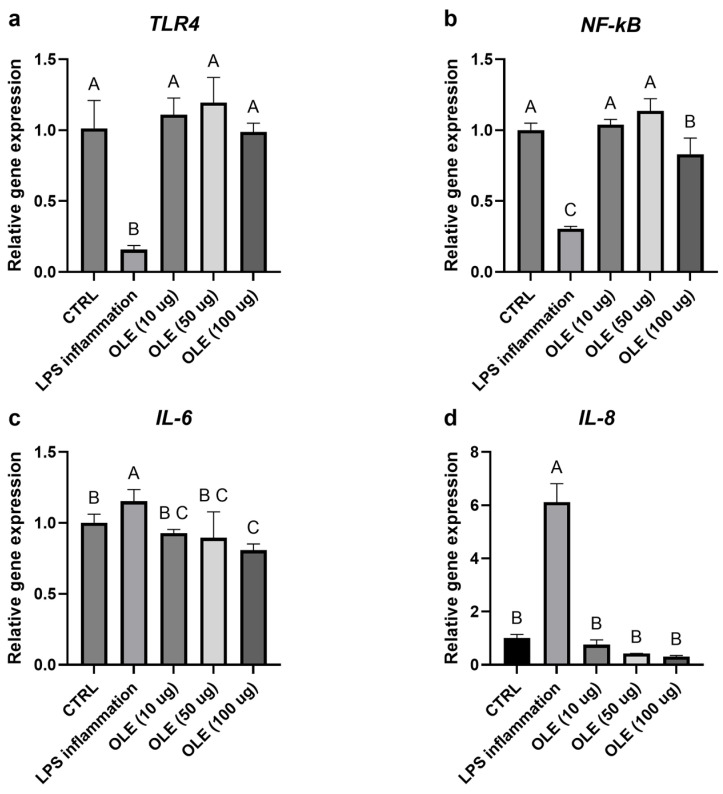
Relative gene expression of TLR4 (**a**), NF-Kb (**b**), IL-6 (**c**) and IL-8 (**d**). Transcript levels were evaluated in cells exposed to LPS and different OLE concentrations (10, 50 and 100 µg/mL). Data are expressed as mean ± S.D (n = 6). Statistical analysis was performed using one-way ANOVA followed by Tukey’s post hoc test. Different letters above the columns represent significant differences among the experimental groups (*p* < 0.05).

Morphological observations were performed to qualitatively support the quantitative cell viability data obtained from the MTT assay. Representative phase-contrast images of SH-SY5Y cells were acquired using an inverted microscope prior to RNA extraction (Figure 3). Control cells and cells treated with 10 and 50 µg/mL OLE showed comparable overall cell density and morphology, consistent with viability values above ~85% measured at the corresponding time points. In contrast, treatment with 100 µg/mL OLE was associated with a visible reduction in cell density and increased cell detachment, in agreement with the decrease in cell viability observed in the MTT assay at longer exposure times. Accordingly, 100 µg/mL was considered a borderline, higher-dose condition and was not used to support assumptions of biological neutrality. No quantitative morphometric analysis was performed; therefore, Figure 3 is presented as supportive visual evidence complementing quantitative viability measurements, rather than as an independent quantitative or mechanistic endpoint. Following image acquisition, cells were harvested and processed for RNA extraction and quantitative RT-PCR analysis of inflammation- and oxidative stress-related genes.

### 3.5. Effects on Inflammation and Oxidative Stress (qPCR)

**Figure 4:**
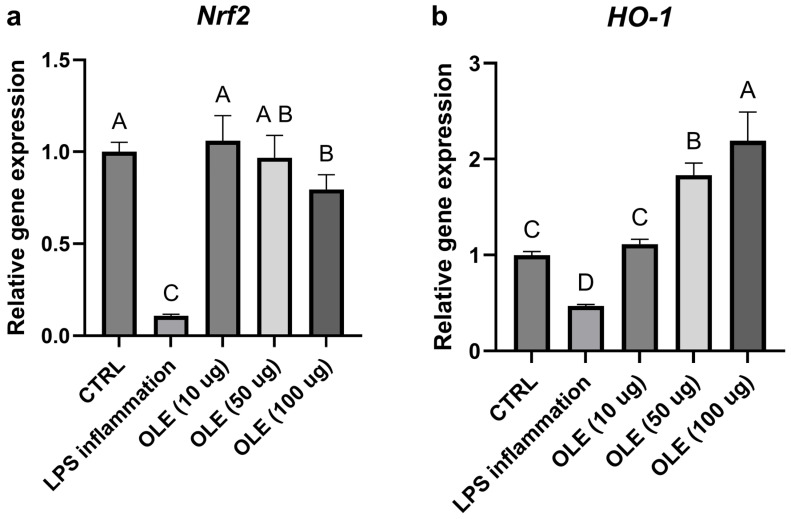
Relative gene expression of Nrf2 (**a**) and Ho-1 (**b**). Transcript levels were evaluated in cells exposed to LPS and different OLE concentration (10, 50 and 100 µg/mL). Data are expressed as mean ± S.D (n = 6). Statistical analysis was performed using one-way ANOVA followed by Tukey’s post hoc test. Different letters above the columns represent significant differences among the experimental groups (*p* < 0.05).

**Figure 5:**
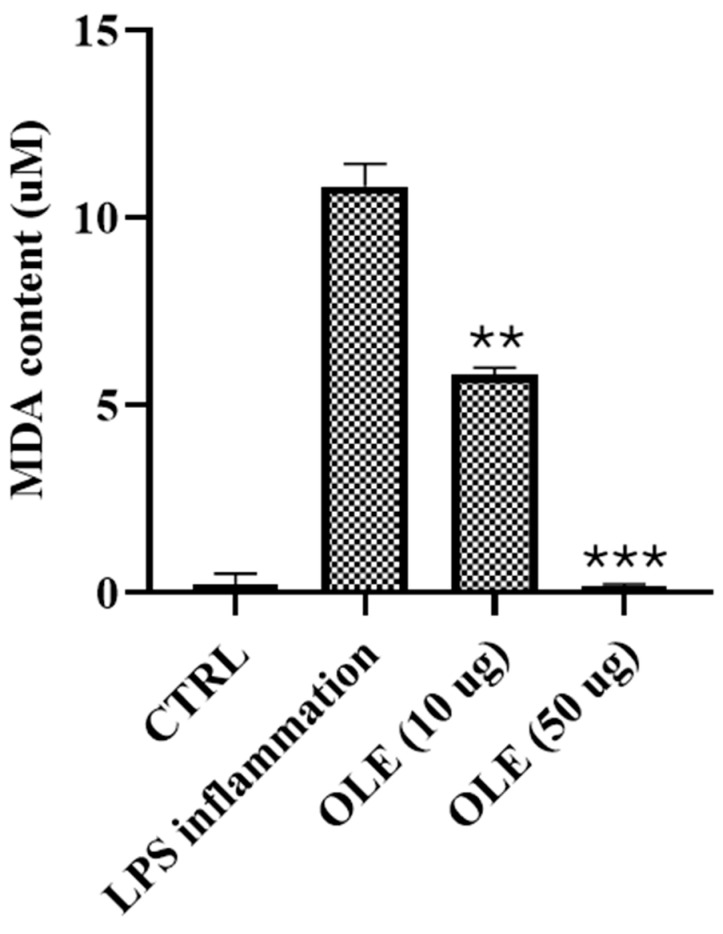
Malondialdehyde (MDA) levels in SH-SY5Y cells exposed to LPS and treated with OLE. Data are mean ± SD of three independent experiments. Data are presented as mean ± SD. Statistical analysis was performed using one-way ANOVA followed by Bonferroni post hoc test (** *p*-values < 0.01, *** *p*-values < 0.001).

Exposure of SH-SY5Y neuroblastoma cells to LPS markedly altered the expression of genes involved in inflammatory and antioxidant pathways. As shown in Figure 4a, b, TLR4 and NF-κB mRNA levels were reduced compared with the untreated control, consistent with a late-phase or adaptive transcriptional response following prolonged LPS exposure. In parallel, *Nrf2* and its downstream effector HO-1 were also downregulated in the LPS-treated group, suggesting that prolonged inflammatory stress may have suppressed the cellular antioxidant defense system (Figure 5a,b). Treatment with the olive-leaf extract (OLE) following LPS removal was associated with dose-dependent changes in gene expression. TLR4 and NF-κB transcript levels increased relative to the LPS group, approaching control values. At the same time, *Nrf2* and *HO-1* were significantly up-regulated, indicating activation of the antioxidant and cytoprotective response. In contrast, the pro-inflammatory cytokines IL-6 and IL-8, which were strongly induced by LPS, showed a marked concentration-dependent reduction upon OLE treatment, with the highest extract concentration restoring values close or even lower to those of the untreated control (Figure 4c,d). Overall, these data show that olive-leaf extract is associated with changes in the expression of inflammation- and redox-related genes following LPS exposure, including reduced IL-6 and IL-8 transcripts and increased Nrf2 and HO-1 expression. Due to the absence of kinetic analyses and co-treatment conditions, these transcriptional changes should be interpreted as indicative of a general modulatory effect on cellular inflammatory and redox status rather than as direct evidence of selective pathway targeting. Given the morphological alterations observed at 100 µg/mL, results at this concentration were interpreted cautiously as a higher-dose condition rather than evidence of biologically neutral exposure.

### 3.6. Lipid Peroxidation: Quantification of Malondialdehyde (MDA)

Exposure of SH-SY5Y cells to lipopolysaccharide (LPS) resulted in a marked increase in malondialdehyde (MDA) levels, reaching 10.83 µM and confirming the induction of a strong oxidative condition. Treatment with *Olea europaea* L. cv. Ogliarola leaf extract (OLE) significantly attenuated lipid peroxidation in a dose-dependent manner. Administration of 10 µg/mL reduced MDA levels to 5.82 µM, while 50 µg/mL almost completely abolished peroxidation, restoring values close to baseline as shown in Figure 5.

These findings highlight the robust antioxidant activity of OLE in protecting cellular membranes from oxidative lipid damage, in line with the well-documented properties of olive polyphenols, which mitigate oxidative stress through direct scavenging of reactive oxygen species and activation of endogenous defense pathways. MDA measurements were performed using OLE concentrations (10 and 50 μg/mL) selected on the basis of the MTT assay to ensure a fully within the no-overt-cytotoxicity range and to minimize potential confounding effects related to reduced cell viability. Although 100 μg/mL was within the no-overt-cytotoxicity range at 24 h, this concentration showed a clear viability decline at longer exposure times and was therefore not included in the lipid peroxidation assay to avoid ambiguous interpretation of antioxidant effects. MDA levels are reported as absolute concentrations (μM). Although all experimental groups were seeded at the same initial cell density and processed at the same experimental time-point, we acknowledge that treatment-related differences in cell viability, metabolism, and morphology may result in variations in final cell number. Therefore, MDA values are interpreted as comparative indicators of lipid peroxidation under controlled conditions, rather than as fully cell-normalized quantitative measures.

## 4. Discussion

Olive tree residues, including leaves, represent an under-utilized renewable resource in Mediterranean regions, with increasing interest for their valorization into high-value nutraceutical products within a circular economy framework [38,39,40]. In this context, olive leaves are particularly attractive due to their high content of bioactive phenolic compounds, that are proven to explicit neuroprotective effects in neuronal models, as the SH-SY5Y in vitro model [38,39,40]. Conversion routes encompass thermochemical, biochemical, and chemical processes leading to the production of bioethanol, biomethane, biodiesel, bioplastics, biochar, and activated carbon [41,42]. In this context, leaves from the cultivar Ogliarola were selected as a model of circular economy valorization for nutraceutical development. This study was designed as a late-phase transcriptional analysis following LPS removal; therefore, the results should be interpreted as modulatory and adaptive cellular responses rather than evidence of direct pathway targeting. The Ogliarola leaf extract exhibited a high total phenolic content, dominated by oleuropein and accompanied by hydroxytyrosol, verbascoside, and luteolin/apigenin derivatives, a profile consistent with previous olive-leaf analyses [43]. The abundance of oleuropein, hydroxytyrosol, verbascoside, and flavonoid derivatives provides a mechanistic rationale for the antioxidant and anti-inflammatory effects observed, given the well-established redox activity of these phenolic structures [44]. In particular, the high abundance of oleuropein and hydroxytyrosol identified in the Ogliarola leaf extract (Table 1) provides a molecular framework to interpret the transcriptional responses observed in SH-SY5Y cells. Oleuropein and its metabolites, including hydroxytyrosol, have been widely associated with antioxidant responses reported for olive polyphenols.

Within this context, the increased expression of Nrf2 and HO-1 and the concomitant reduction in IL-6 and IL-8 transcripts observed after treatment with the Ogliarola extract are consistent with the known molecular actions of oleuropein-rich olive leaf matrices, although direct pathway activation was not experimentally addressed in the present study.

Such structure and activity relationships provide a mechanistic rationale for the antioxidant and anti-inflammatory effects observed in our study. The predominance of oleuropein, accounting for approximately 78% of the quantified phenolic content, represents a distinctive chemical feature of the Ogliarola cultivar and supports its selection for nutraceutical valorization. This cultivar-specific enrichment strengthens the link between chemical composition and the cellular responses observed in SH-SY5Y cells. In the human neuroblastoma SH-SY5Y model, the *Olea europaea* L. cv. Ogliarola leaf extract (OLE) was within the no-overt-cytotoxicity range at concentrations up to 100 µg/mL after 24 h, while the safe window narrowed to ≤50 µg/mL at 48 h. This dose- and time-dependent behavior mirrors findings reported for olive phenolics and polyphenol-rich extracts in neuronal models, where low-to-moderate concentrations are generally well tolerated, whereas higher doses or prolonged exposures can reduce viability [45]. However, most previous studies have focused on mixed olive leaf extracts or on cultivars other than Ogliarola, often reporting variable phenolic compositions and bioactivities. The high oleuropein content and the marked antioxidant response observed in the present study suggest that the Ogliarola cultivar may display a particularly favorable bioactive profile compared to other reported varieties, although direct quantitative comparisons remain limited by differences in extraction protocols and experimental models.

Defining this within the no-overt-cytotoxicity range ensures that the bioactivities subsequently measured such as antioxidant or antiglycation effects reflect genuine protective mechanisms rather than secondary consequences of cytotoxicity. The down-regulation of *TLR4* and *NF-κB* transcripts observed after 24 h of LPS exposure may represent the resolution phase of the inflammatory response. Importantly, this increase should not be interpreted as evidence of beneficial anti-inflammatory pathway activation or targeted modulation of TLR4/NF-κB signaling, but rather as a potential indicator of general cellular recovery following prolonged inflammatory stress and LPS removal. During the early hours of stimulation, LPS strongly activates the TLR4–NF-κB axis, triggering the transcription of pro-inflammatory mediators such as IL-6 and IL-8. Prolonged exposure, however, induces feedback inhibitory mechanisms, which collectively suppress further transcription of *TLR4* and *NF-κB* subunits, explaining their lower mRNA levels at 2 days of incubation [46,47]. It should be emphasized that the LPS model used in this study does not assess acute inflammatory signaling or direct pathway inhibition, but rather reflects a late-phase cellular condition following prolonged inflammatory stress and LPS removal. The subsequent treatment with olive-leaf extract (OLE) promoted a partial recovery of *TLR4* and *NF-κB* expression, likely reflecting the changes in gene expression of basal homeostatic signaling. At the same time, OLE strongly enhanced the expression of *Nrf2* and *HO-1*, two key components of the antioxidant defense network. The phenolic compounds oleuropein and hydroxytyrosol, abundant in olive leaves, are known to activate Nrf2, leading to Nrf2 nuclear translocation and transcriptional activation of cytoprotective genes such as *HO-1* [44]. The concurrent up-regulation of *Nrf2/HO-1* and down-regulation of *IL-6* and *IL-8* indicates that OLE shifts the cellular state from an inflammatory to an antioxidant and neuroprotective phenotype. This dual modulatory effect restoring physiological *NF-κB/TLR4* expression while enhancing antioxidant defenses aligns with previous evidence showing that olive polyphenols inhibit cytokine production while stimulating Nrf2-dependent antioxidant enzymes [45,46]. OLE modulated the transcription of genes associated with inflammatory and antioxidant pathways, promoting a shift toward a less pro-inflammatory and more cytoprotective transcriptional profile. It is important to underline that the present experimental design focused on a single late time point following LPS exposure. Accordingly, the observed modulation of TLR4-, NF-κB-, Nrf2-, and HO-1-related transcripts should be interpreted as late-phase modulatory trends rather than direct mechanistic evidence of selective pathway activation or inhibition. These transcriptional trends are consistent with previously reported neuroprotective mechanisms of olive-derived phenolics. In particular, hydroxytyrosol has been shown to suppress TLR4-mediated NF-κB activation and downstream ERK signaling in LPS-induced neuroinflammatory models, resulting in attenuation of pro-inflammatory cytokine production and oxidative stress. Similar effects have been described for oleuropein and its metabolites, which modulate microglial and neuronal inflammatory signaling while enhancing antioxidant defenses. Within this framework, the gene-expression changes observed in SH-SY5Y cells following treatment with the Ogliarola leaf extract align with established molecular actions of olive polyphenols and support its potential translational relevance as a neuroprotective nutraceutical ingredient. LPS exposure in SH-SY5Y cells markedly increased malondialdehyde (MDA) levels (10.83 µM), confirming the induction of oxidative stress. Treatment with OLE significantly decreased MDA to 5.82 µM at 10 µg/mL, and nearly restored baseline levels at 50 µg/mL. These results are consistent with prior studies showing that olive phenolics suppress lipid peroxidation by scavenging reactive oxygen species and enhancing endogenous antioxidant defenses [48]. The dose-dependent reduction in MDA observed here therefore supports the strong antioxidant potential of the Ogliarola leaf extract. This antioxidant activity is likely linked to Nrf2-driven transcriptional activation of detoxifying enzymes, which can decrease lipid peroxidation by increasing the cellular capacity to neutralize ROS and repair oxidative damage. The extract inhibited advanced glycation end-product (AGE) formation by ~31.5% in the BSA–MGO model, consistent with previous reports showing that olive-leaf phenolics attenuate protein glycation [49].

Given the central role of AGEs in diabetes-related and neurodegenerative disorders, this antiglycation activity complements the antioxidant response and broadens the functional profile of OLE as a food-derived bioactive with dual antioxidant and antiglycation potential. Given the reciprocal relationship between glycoxidation and inflammation, the observed antiglycation effect may also contribute indirectly to the attenuation of pro-inflammatory signaling, suggesting a broader protective action on neuronal homeostasis. Notably, to the best of our knowledge, this study represents the first evaluation of an Ogliarola olive leaf extract integrating oxidative stress, lipid peroxidation, antiglycation activity, and inflammatory responses in a neuronal cell model. Collectively, the Ogliarola extract modulated interconnected pathways of oxidative stress, lipid peroxidation, and glycation within a within the no-overt-cytotoxicity range window in SH-SY5Y cells. This cultivar-specific approach is relevant because phenolic content and composition vary widely among olive leaves, influencing bioactivity and extraction yield [50]. Although the present findings derive from an in vitro system, they contribute to the growing body of evidence supporting the nutraceutical relevance of leaf-derived extracts. Future studies should clarify molecular mechanisms in animal models for nutraceutical applications.

## 5. Conclusions

This study demonstrates that phenol-rich olive leaf extract from *Olea europaea* L. cv. Ogliarola modulated multiple oxidative, inflammatory, and glycoxidative endpoints in SH-SY5Y neuronal cells within a non-cytotoxic concentration range. The extract significantly reduced lipid peroxidation and advanced glycation end-product formation, while influencing the transcription of genes associated with inflammatory and antioxidant responses, supporting a cytoprotective cellular profile.

Although these findings derive from an in vitro neuronal model and are based on transcriptional analyses, they provide a solid biochemical and cellular rationale for the valorization of Ogliarola olive leaves as a sustainable source of bioactive compounds for nutraceutical development. Further studies integrating kinetic analyses, protein-level validation, and compound-specific fractionation will be required to clarify pathway-specific mechanisms and translation to more complex biological systems.

## Figures and Tables

**Table 1:** Primers used in this study.

Gene Name	Forward	Reverse	Product Size (bp)	Reference
***IL-6***	AGACAGCCACTCACCTCTTCAG	TTCTGCCAGTGCCTCTTTGCTG	131	[32]
***IL-8***	TGAGAGTGATTGAGAGTGGACC	ACTTCTCCACAACCCTCTGC	120	This study
***TLR4***	TATCAGAGCCTAAGCCACCT	ATTTGTCTCCACAGCCACC	120	This study
***HO-1***	TGCCAGTGCCACCAAGTTCA	GATGTTGAGCAGGAACGCAG	118	This study
***NF-kB***	AAGCAGGAAGATGTGGTGGAG	CGTTGGGGTGTCAAGAAGTAGT	169	This study
***Nrf2***	CAGCGACGGAAAGAGTATGA	TGGGCAACCTGGGAGTAG	200	[33]
***GAPDH***	GGAAGGTGAAGGTCGGAGTC	TGGAAGATGGTGATGGGATTT	174	This study

Amplification efficiency of all qPCR reactions was within 90–110%.

**Table 2:** Content (mg kg^−1^) of phenol compounds in hydroalcoholic olive leaf extract analyzed. Relative standard deviation (RSD) values for all the quantified compounds were under the 8%.

N°	Phenolic Compound	Concentration
1	Gallic acid	149.7 ± 5.2
2	Hydroxytyrosol glucoside ^a^	1514.1 ± 14.0
3	Hydroxytyrosol	786.5 ± 9.3
4	Oleoside ^b^	2065.1 ± 17.9
5	Hydroxyoleuropein ^b^	4792.6 ± 33.0
6	Verbascoside	3274.0 ± 29.4
7	Luteolin 7-O-glucoside ^c^	2954.3 ± 22.6
8	Oleuropein glucoside ^b^	2822.8 ± 15.9
9	Apigenin rutinoside ^d^	239.1 ± 4.1
10	Apigenin glucoside ^d^	2240.3 ± 16.3
11	Luteolin 4-O-glucoside ^c^	789.8 ± 11.2
12	Oleuropein	83,331.8 ± 61.5
13	Lucidumoside C ^b^	1868.2 ± 18.2
	***Tot.***	***106,828.3 ± 246.6***

Bioactive molecules were quantified based on calibration curves of the correspondent standard compounds: ^a^ hydroxytyrosol, ^b^ oleuropein, ^c^ luteolin, ^d^ apigenin.

## Data Availability

The original contributions presented in this study are included in the article/Supplementary Materials. Further inquiries can be directed to the corresponding author.

## Supplementary Materials

The following supporting information can be downloaded at: https://www.mdpi.com/article/10.3390/foods15010043/s1, Table S1. Regression equations, correlation coefficients (*R*^2^), LOD, LOQ, recoveries, retention times, *λ*_max_ and MS values for each bioactive molecule. Intraday and interday repeatability were evaluated at one concentration level (1 mg/L), and showed coefficient of variation (CV) values <7.5 %. All the compounds analysed showed a resolution value > 1.2.
