# Supplementary material for: Chemical Characterization of Phenol-Rich Olive Leaf Extract (Olea europaea L. cv. Ogliarola) and Its Neuro-Protective Effects on SH-SY5Y Cells from Oxidative Stress, Lipid Peroxidation, and Glycation
Source: Foods. 2025 Dec 23;15(1):43. doi: 10.3390/foods15010043 (PMC12786155; doi:10.3390/foods15010043)
Supplement: Supplementary file 1 [file foods-15-00043-s001.zip › foods-4027897-supplementary.pdf]

Table S1. Regression equations, correlation coefficients (R^2), LOD, LOQ, recoveries, retention times, λ_{max} and MS values for each bioactive molecule. Intraday and interday repeatability were evaluated at one concentration level (1 mg/L), and showed coefficient of variation (CV) values <7.5 %. All the compounds analysed showed a resolution value > 1.2.

**Compound**	**Regression line**	**R^2**	**LOD (mg/L)**	**LOQ (mg/L)**	**Recovery (%)**	**λ_{max}**	***[M-H]$^{-1}$***
Gallic acid	y = 0,0009x + 0,0007	0.993	0.039	0.092	89.4	216, 271	169
Hydroxytyrosol	y = 0,0014x + 0,0039	0.992	0.043	0.0092	87.3	280	153
Verbascoside	y = 0,0043x - 0,0007	0.993	0.035	0.79	90.2	217, 329	623
Oleuropein	y = 0,0087x + 0,0048	0.994	0.046	0.098	80.1	234, 248, 279	539
Luteolin	y = 0,0089x + 0,022	0.994	0.037	0.083	91.0	254, 354	285
Apigenin	y = 0,0127x + 0,063	0.993	0.026	0.078	84.2	266, 336	269
